# Biocide Response of *Candida auris*

**DOI:** 10.1111/myc.70169

**Published:** 2026-03-19

**Authors:** Sidre Erganis, Ali Ozturk, Elif Ayca Sahin, Ayse Kalkanci

**Affiliations:** ^1^ Faculty of Medicine Department of Medical Microbiology Gazi University Ankara Türkiye; ^2^ Faculty of Medicine Department of Medical Microbiology Nigde Omer Halisdemir University Nigde Türkiye

**Keywords:** biocide activity tests, biocide tolerance, *Candida auris*, chlorine‐based disinfectants, disinfectant resistance, environmental persistence, infection control, nosocomial transmission, quaternary ammonium compounds

## Abstract

*Candida auris* is an emerging multidrug‐resistant yeast demonstrating remarkable persistence in healthcare environments, contributing to nosocomial transmission and outbreak persistence. Increasing disinfectant failure reports have raised concerns regarding infection control policies, as environmental reservoirs play central roles in its spread. We reviewed experimental studies, environmental surveillance reports, and comparative disinfection efficacy data to summarise interactions between *C. auris* and commonly used biocidal classes: chlorine‐based oxidizers, alcohol formulations, biguanides, and quaternary ammonium compounds. Mechanistic findings on biofilm formation, efflux activity, and stress‐response pathways were integrated to contextualise tolerance behaviour. Evidence indicates *C. auris* shows reduced susceptibility to quaternary ammonium compounds and demonstrates variable, strain‐dependent tolerance to alcohol‐based disinfectants, particularly with organic load or suboptimal contact times. Chlorine‐based oxidising agents maintain reliable activity at appropriate concentrations and exposure durations. Biofilm formation enhances environmental persistence and diminishes surface decontamination efficiency. *C. auris* requires disinfectant strategies distinct from other *Candida* species. Effective infection prevention depends on optimised agent selection, adequate contact times, and consideration of surface and organic‐matter conditions. Tailored decontamination protocols are essential to limit environmental persistence and interrupt nosocomial transmission.

**Abbreviations**
AOAC: association of official analytical cooperation
ASTM: American society for testing and materials
ATCC: American type culture collection
BAT: biocide activity test(s)

*C. auris*

: *candida auris*
CDC: centres for disease control and prevention
CLSI: clinical and laboratory standards institute
ECOFF: epidemiological cutoff value
EN: European norm
EPA: environmental protection agency (USA)
H₂O₂: hydrogen peroxide
MIC: minimum inhibitory concentration
NaOCl: sodium hypochlorite
PAA: peracetic acid
QAC(s): quaternary ammonium compound(s)
UV‐C: ultraviolet C

## 1 General Biological Background of *Candida auris*

### 1.1 Taxonomy and Emergence

*Candida auris (Syn. Candidozyma auris)* is an opportunistic yeast belonging to the phylum Ascomycota, class Saccharomycetes, and family Metschnikowiaceae. It was first described in 2009 from the external ear canal of a patient in Japan, although retrospective analyses have traced earlier isolates back to the late 1990s in South Korea. The species name ‘auris’ derives from its original site of isolation (Latin: ear). In 2024, phylogenomic analyses led to its reclassification, but the current consensus is to keep using *Candida auris*.

Epidemiologically, *C. auris* is divided into six major clades with strong geographic signatures (Table 1).

**TABLE 1:** Clade‐specific characteristics and antifungal resistance profiles of *Candida auris*.

Clade	Origin	Key phenotypic/genotypic characteristics	Azoles	Echinocandins	Amphotericin B	MDR profile
Clade 1	South Asia	High outbreak potential; strong environmental persistence; robust biofilm formation; frequent **ERG11** and **TAC1B** mutations	Resistant (fluconazole common)	Variable; **FKS1** mutations reported	Variable‐reduced susceptibility	Common
Clade 2	East Asia	Mainly ear isolates; low outbreak potential; limited environmental spread	Generally susceptible/low MICs	Susceptible	Susceptible	Rare
Clade 3	Africa	Associated with invasive infections; moderate outbreak potential	Frequently resistant	Mostly susceptible	Variable	Reported
Clade 4	South America	High environmental persistence; strong healthcare‐associated transmission; genetically closest to Clade VI	Resistant	Mostly susceptible	Reduced susceptibility	Common
Clade 5	Iran	Genetically distinct; limited number of isolates; epidemiology not fully defined	Variable	Susceptible	Variable	Uncommon
Clade 6	Indomalayan	Newly identified clade; distinct chromosomal rearrangements; different mating‐type alleles; growth at 42°C	Susceptible	Susceptible	Susceptible	Not observed

Its global emergence has been linked to climate change, antifungal overuse and environmental adaptation, underscoring its ‘One Health’ relevance [1, 2, 3].

### 1.2 . Morphology and Growth

*C. auris* is an oval yeast cell typically measuring 2–5 μm, reproducing by budding. Unlike *Candida albicans* , it does not readily form true hyphae, though some isolates generate pseudohyphae‐like structures under stress. Rarely, true hyphae have been induced under special conditions [4, 5]. Colony morphology varies on CHROMagar, ranging from white to pink or purple, often influenced by environmental stress [5].

The yeast grows optimally at 37°C–40°C, and some strains tolerate up to 42°C. Remarkably, it also thrives under high salinity (> 10% NaCl), reflecting strong thermotolerance and halotolerance. These traits not only facilitate survival in diverse niches but also enhance persistence on hospital surfaces [6].

### 1.3 Genomic Features

The haploid genome is approximately 12–13 Mb, encoding over 8500 genes. Its mitochondrial genome is about 27 kb, containing standard rRNA, tRNA, and protein‐coding loci. A hallmark genetic feature is the alternative CTG codon usage, translating serine instead of leucine, a property shared with other members of the *Candida haemulonii* species complex.

Whole‐genome sequencing has demonstrated high inter‐clade diversity with tens of thousands of SNPs between clades, while intra‐clade isolates are relatively clonal—helping explain outbreak dynamics [7, 8].

### 1.4 Reproduction and Ecology

*C. auris* reproduces primarily by budding in yeast form; no sexual or parasexual cycle has been confirmed, though genomic data suggest the presence of mating‐type loci. Ecologically, the fungus is hypothesized to have originated from wetland environments or plant‐associated niches, later adapting to humans via thermotolerance and antifungal resistance mutations. Migratory birds and human movement are proposed to have facilitated its global spread [9].

### 1.5 Virulence Traits

Although *C. auris* is not inherently the most virulent species within the genus *Candida*, it possesses a distinctive combination of traits that collectively enable persistent colonisation and opportunistic infection. Among these, adhesion molecules—including orthologs of the ALS family—facilitate binding to both host tissues and abiotic surfaces, while its capacity for biofilm formation, though yielding structurally thinner biofilms compared to *C. albicans* , plays a critical role in survival on skin and hospital surfaces alike. In addition, *C. auris* secretes a repertoire of hydrolytic enzymes, such as aspartyl proteinases, phospholipases, and hemolysins, which collectively support tissue invasion and immune evasion. These virulence attributes are further complemented by notable thermotolerance and osmotolerance, properties that allow the organism to persist across a wide range of host and environmental conditions. Taken together, *C. auris* emerges as a thermotolerant, halotolerant, budding yeast characterised by remarkable genomic plasticity and ecological adaptability. It is this unique biological profile that underpins its ability to endure in hospital environments and disseminate across continents, positioning it as a singular model of fungal emergence [10, 11].

## 2 Biocide Activity Tests for *Candida auris* Susceptibility

Biocide Activity Tests (BAT) were developed to evaluate the effectiveness of biocidal agents under standard conditions. Internationally recognised test protocols are defined by European Norms (EN), the American Society for Testing and Materials (ASTM), and the Association for Official Analytical Cooperation (AOAC) [12].

### 2.1 Suspension Tests

According to the EN 13624 standard, these are used to determine the effectiveness of a particular product, its fungicidal/bactericidal activity by measuring the logarithmic decrease of the organism in question under standardised conditions (constant concentrations, contact times, etc.). Studies on *C. auris* have reported low log reductions with chlorhexidine and alcohol‐based agents, whereas high log reductions were observed with oxidising agents [13].

#### 2.1.1 Surface Tests

The EN 13,697 standard evaluates the effectiveness of biocides on glass, stainless steel, or plastic surfaces. Chlorine‐based agents provide a ≥ 5 log10 reduction, while QACs generally fail to reach this level [14, 15]. Importantly, for chlorine‐based disinfectants, fungicidal efficacy under EN 13,697 conditions is highly dependent on the concentration of available chlorine and exposure time, with suboptimal concentrations frequently resulting in insufficient log reductions (14). In contrast, reports describing sufficient activity of QACs are often based on test standards that include defined mechanical action (e.g., EN 16115‐based wipe tests), which is not incorporated into EN 13,697 surface tests [15]. This methodological distinction is critical when interpreting discrepant efficacy results across studies.

#### 2.1.2 Carrier and Capacity Tests

Microorganisms inoculated onto solid surfaces are dried and treated with biocides. In capacity tests, effectiveness is measured in the presence of an organic load (blood, serum). *C. auris* biofilms significantly reduce biocide activity under these conditions [13, 16].

### 2.2 Practical (Field) Tests

These tests, which simulate clinical conditions, reflect the performance of the biocide in a real environment. The fact that some biocides are effective in laboratory settings but fail in the field during outbreaks demonstrates the importance of these tests [12, 13, 16].

### 2.3 International Standards and Test Methodologies

The reference framework for BAT is provided by EN 14885, EN 13624, EN 13,697 and ASTM E2197 [10, 11]. However, these standards are mostly designed based on reference species such as *C. albicans* ATCC 10231. Given the biofilm‐forming capacity, antifungal resistance and long‐term survival on surfaces of *C. auris* , existing protocols need to be updated specifically for this pathogen [17].

While EN standards include fungicidal and yeasticidal activity assessments, *C. auris* is not included as a reference microorganism. ASTM E2197 is based on *Staphylococcus aureus* ATCC 6538 and *C. albicans* ATCC 10231 but does not include *C. auris* [12, 13, 16].

In recent years, the Environmental Protection Agency (EPA) and Centres for Disease Control and Prevention (CDC) have developed specific methodologies to validate disinfectant claims for drug‐resistant *C. auris* [17, 18, 19]. The EPA has published laboratory guidelines for labelling ‘ *C. auris* ‐approved’ biocides, and the CDC has recommended their use in outbreak control.

The limitations of current standards relate not only to the test microorganisms but also to the exclusion of biofilm models. Current recommendations emphasise the inclusion of *C. auris* biofilms, along with planktonic cultures, in validation tests [20].

### 2.4 Implications for Clinical Practice and Infection Control

*Candida auris* poses serious challenges in infection control because it can survive for 7–14 days on plastic, stainless steel, textile and wall surfaces [21]. Many biocides used in routine cleaning are reported to be inadequate against this pathogen [22]. The CDC has reported that some common disinfectants are ineffective and recommends the use of only EPA‐approved biocides [17]. QAC‐based products fail to remove contamination, whereas chlorine‐based sporicidal agents, peracetic acid (PAA), and hydrogen peroxide (H_2_O_2_) provide ≥ 5 log10 activity [23].

Biocide efficacy may be reduced in the presence of organic matter (such as blood, serum, or mucus) [21]. Therefore, the use of appropriate biocide combinations in conjunction with mechanical cleaning is recommended in high‐risk areas such as intensive care and haematology‐oncology wards. The CDC also emphasizes the importance of patient isolation, the use of disposable supplies, device decontamination, and terminal cleaning practices [24].

In recent years, UV‐C irradiation and vapour‐based H_2_O_2_ systems have emerged as adjunct methods to biocides [8]. Current approaches indicate that the use of combined or nanoparticle‐supported formulations instead of single agent biocides can enhance efficacy by increasing biofilm penetration [25].

## 3 Susceptibility of *Candida auris* to Biocides Used in Hospitals

*Candida auris* ( *C. auris* ) studies to assess the efficacy of biocides include laboratory‐based minimum inhibitory concentration (MIC) measurements and quantitative activity tests as mentioned above simulating surface conditions [26, 27]. The broth microdilution test (adapted from CLSI M27‐A2) is performed in microtiter plates, similar to antifungal susceptibility tests. The MIC value is determined as the lowest biocide concentration that inhibited visible growth by at least 50% [12, 13, 28]. This method was used to determine the susceptibility limits of *C. auris* to different biocides.

Biocides form the basis of surface disinfection and infection control in hospital settings. Among the most frequently preferred agents are chlorine compounds (sodium hypochlorite), chlorhexidine, benzalkonium chloride, and triclosan [12, 13]. Alcohol‐based hand antiseptics (especially 70% ethanol) are also used as complementary biocides for both hand hygiene and the decontamination of certain surfaces [13].

### 3.1 Chlorine Compounds (Sodium Hypochlorite)

Sodium hypochlorite (NaOCl) is considered the standard biocide for hospital disinfection due to its effectiveness against *C. difficile* spores [16]. Chlorine‐based disinfectants, especially in 500 ppm solutions, exhibit broad‐spectrum antimicrobial activity and are widely used for hospital surface cleaning [13]. Surfaces should be cleaned with soap and water before using a chlorinated cleaning product. *C. auris* strains are known to have variable tolerance to low‐concentration chlorine solutions [13]. In particular, it has been shown that 1–5 min of exposure to a 200 ppm chlorine solution does not provide an effective logarithmic decrease and does not produce a fungicidal effect. However, exposure for up to 30 min is partially effective [13]. In contrast, a chlorine concentration of 500 ppm successfully combated Clade 1 *C. auris* from the first minute in a polluted environment but proved ineffective against Clade 4 *C. auris* [13].

### 3.2 Chlorhexidine

Chlorhexidine is a biguanide compound widely used in the surgical field for antisepsis and skin decontamination. Studies have found MIC values for chlorhexidine in *C. auris* strains between 0.5–1 mg/L [13]. These values are close to the epidemiological cutoff of 2 mg/L, indicating borderline susceptibility [28]. Chlorhexidine tolerance has increased in some isolates, particularly in strains that form dense biofilms [10]. Furthermore, solutions containing 2% chlorhexidine were insufficient to provide logarithmic reductions in contaminated surface conditions; however, 4% chlorhexidine solutions were found to be effective [13]. Consequently, it is crucial to use a high concentration of at least 4% chlorhexidine with sufficient contact time to disinfect *C. auris* .

### 3.3 Benzalkonium Chloride

Benzalkonium chloride is a quaternary ammonium compound (QAC) widely used for surface disinfection. Although microdilution‐based biocide susceptibility assays indicate that benzalkonium chloride possesses strong theoretical antimicrobial activity, high‐level resistance has been consistently reported in *C. auris* isolates [13, 26]. The Epidemiological cutoff value (ECOFF) for benzalkonium chloride is 32 mg/L, and the MIC values of *C. auris* strains typically range between 16 and 32 mg/L [29]. These findings confirm the poor susceptibility of *C. auris* to quaternary ammonium‐based disinfectants. Moreover, environmental parameters such as contact time and surface cleanliness were shown to markedly reduce its efficacy [12, 13, 28]. Therefore, benzalkonium chloride is considered ineffective for *C. auris* decontamination and is not recommended for use in clinical practice.

### 3.4 Triclosan

Triclosan is a phenolic derivative found in antiseptic soaps and some surface disinfectants. Triclosan MIC values for *C. auris* isolates were determined to range between 0.0078 to 0.5 mg/L [12]. The ECOFF value is considered to be 0.5 mg/L [29]. These findings indicate that triclosan may be effective at low concentrations, but tolerance may develop in strains with high MIC values.

### 3.5 Alcohol‐Based Antiseptics

Alcohol‐based surface disinfectants are frequently used in healthcare settings for hand antisepsis and environmental surface cleaning. Ethyl alcohol, in the 40%–70% concentration range, meets the ≥ 4 log10 reduction criterion for yeast efficacy according to the EN 13624 standard [16]. Antiseptics containing 70% ethanol exhibit short‐term fungicidal activity against *C. auris* . Our study revealed a full logarithmic reduction was achieved after 1 min of exposure in clinical isolates from Clade 1 *C. auris* [13]. However, the surface efficacy of alcohol‐based disinfectants appears to be variable and clade‐dependent. In a recent study by Lang et al., ethanol‐based surface disinfectants evaluated using standardised carrier‐based surface tests demonstrated heterogeneous activity across different *C. auris* clades, with some isolates failing to reach the ≥ 4 log10 reduction threshold under practical surface conditions [16]. However, in the EN 16615 four‐field test, the effectiveness of alcohol‐based wipes in ‘dirty conditions’ was not sufficient for all clades, meeting the standard only for Clade II isolates [16]. However, alcohol‐based solutions alone are not enough to remove persistent surface contamination; a combination with high‐concentration chlorine and chlorhexidine is recommended for surface cleaning [13].

### 3.6 Cross Resistance of Biocides and Antifungals

Recent studies have shown a phenotypic relationship between biocide susceptibility and antifungal susceptibility. We found a positive correlation (ρ = 0.35) between amphotericin B MIC values and triclosan MIC values [12]. Similarly, anidulafungin MIC was determined to be the parameter that best predicted biocide susceptibility [28]. Our analyses using the Random Forest model, anidulafungin MIC was followed by amphotericin B and flucytosine MICs [28]. These findings suggest that antifungal MIC profiles can be used to predict biocide susceptibility and that this method may be a practical predictive tool for *C. auris* until standardisation of biocide tests is achieved. However, the fact that all of the limited number of clinical strains used were from clade 1, that only one strain of *C. auris* was used as a reference, and that reference strain was clade 4, are major limitations of this study. It does not provide information about other strains.

### 3.7 Virulence Factors in Relation to Biocide Susceptibility

Our studies revealed Clade 1 *C. auris* strains have strong biofilm formation (95%) and SAP activity (100%) which were demonstrated in almost all strains [12, 13, 28]. Esterase activity was detected in 87%, while phospholipase activity was not observed. Caseinase activity was detected in only 4%. An increase in chlorhexidine and triclosan MIC values was observed, especially in strains that formed dense biofilms; this suggested that biocide tolerance may be related to the biofilm‐derived protective barrier [12].

## 4 Biocide Resistance and Environmental Persistence of *Candida auris*

Environmental surfaces in healthcare facilities are increasingly recognised as reservoirs for *Candida auris* and other *Candida* spp. [30]. Recent studies have demonstrated that *C. auris* can survive on dry surfaces for several weeks, withstand exposure to certain disinfectants such as QACs, and form biofilms that further reduce susceptibility to biocides [31, 32, 33]. Effective disinfection strategies therefore require not only chemical potency but also optimised contact times, mechanical cleaning, and an understanding of the microbe's adaptive biology [34]. Recent evidence indicates that *C. auris* exhibits variable susceptibility to commonly used disinfectants, making environmental decontamination a major challenge. NaOCl remains one of the most effective agents, showing complete killing of *C. auris* on different surfaces at concentrations around 1% and contact times of 5–10 min [14]. H_2_O_2_, either as vapour (8 g/m^3^) or solution (0.5%–1.4%), has demonstrated comparable efficacy to chlorine‐based disinfectants, achieving 96%–100% reduction in viable cells. Combinations of PAA, acetic acid, and H_2_O_2_ have also shown synergistic fungicidal activity [35, 36, 37]. In contrast, QACs are largely ineffective against *C. auris* . Other agents such as phenol (5%) and glutaraldehyde (2%) are effective but require prolonged exposure times [37]. Adjunctive physical methods, including UV‐C irradiation and ozone‐based disinfection, can further enhance decontamination efficiency when combined with standard cleaning protocols. Overall, both chemical potency and optimised contact duration are critical for effective *C. auris* eradication from healthcare environments [34, 38].

Building on these findings, the following section focuses on elucidating the mechanisms behind the observed biocide resistance in *C. auris* .

### 4.1 Biocide Resistance Mechanisms of *Candida auris*

Environmental persistence of *C. auris* is closely linked to its resistance mechanisms against commonly used disinfectants and antiseptics. Biofilm formation, stress tolerance, and specific surface adaptations collectively enable the organism to withstand chemical and physical cleaning procedures, contributing to its successful survival and spread within healthcare environments. Surface adhesion represents a key initial step in the biofilm formation and skin colonisation process of *C. auris* . Similar to *C. albicans, C. auris* expresses adhesin proteins belonging to the agglutinin‐like sequence (ALS) family, which function in attachment and virulence [9]. However, the roles of these adhesins vary across isolates and life cycle stages. Experimental models have shown that ALS5 expression is associated with biofilm formation in isolates exhibiting aggregative growth, whereas strains lacking this phenotype show limited expression. Genetic diversity among clades contributes to this variability; notably, Clade II strains lack several cell wall and ALS‐like genes, which may explain their weaker biofilm formation, lower skin colonisation, and absence in major outbreaks [39]. Further studies identified ALS4 overexpression and gene amplification linked to stronger aggregation and dense biofilms, while replicative aging increases ALS5 expression and cell wall thickness, enhancing surface adhesion [40]. In addition to ALS homologues, *C. auris* expresses a unique adhesin, Scf1, found only in *C. auris* and closely related *C. haemulonii* species. Scf1 expression correlates with adhesion ability, and deletion of SCF1 or IFF4109 reduces fungal burden, skin colonisation, and biofilm formation on medical devices in experimental models [41]. Unlike typical hydrophobic fungal adhesins, Scf1 mediates attachment through cationic surface interactions. Collectively, these findings indicate that diverse adhesins, particularly ALS family members and Scf1, are central to its biofilm‐associated virulence, persistence on surfaces, and capacity to resist disinfection in healthcare environments [42].

Transcriptomic studies have further revealed that *C. auris* biofilms upregulate genes associated with efflux pumps and iron acquisition systems, supporting metabolic adaptation and reduced susceptibility to oxidative or membrane‐active biocides [12]. Moreover, sublethal or repeated biocide exposure has been shown to trigger efflux pump activation and cell wall remodelling, contributing to the development of tolerance mechanisms. Dire et al. investigated the survival of *C. auris* on glass, steel, plastic, wood, and fabric under dry and moist conditions for three weeks and observed that the organism maintained viability on all tested materials, with biofilm formation significantly enhancing persistence. Following daily exposure to sodium dichloroisocyanurate and benzalkonium chloride for 15 days, the MICs increased up to four‐fold, indicating tolerance development. Notably, benzalkonium chloride‐exposed isolates showed reduced ergosterol content and activation of energy‐dependent efflux pumps, suggesting adaptive resistance through membrane modification and enhanced efflux activity [33].

The relationship between resistance to antifungal compounds and tolerance to biocides is largely driven by shared molecular mechanisms that promote cross‐resistance or co‐selection. One of the primary mechanisms underlying this association is the overexpression of ATP‐binding cassette (ABC) transporters, such as **Cdr1** and **Snq2**, as well as Major Facilitator Superfamily (MFS) transporters like **Mdr1**, which are capable of exporting both azole antifungals and structurally diverse biocidal agents, including chlorhexidine, triclosan and quaternary ammonium compounds [43].

In addition, alterations in the ergosterol biosynthetic pathway—particularly mutations or upregulation of **ERG11**—not only contribute to azole resistance but may also modify membrane composition and permeability, thereby reducing susceptibility to membrane‐active biocides [44].

Biofilm‐associated growth further exacerbates this phenomenon, as the extracellular matrix acts as a physical and chemical barrier that sequesters both antifungal agents and disinfectants, while the presence of metabolically altered subpopulations, such as persister cells, contributes to a multidrug‐tolerant phenotype [45].

Importantly, in vitro studies have demonstrated that prolonged exposure to sub‐inhibitory concentrations of biocides, including chlorhexidine and triclosan, can impose selective pressure favoring the emergence of azole‐resistant Candida strains, underscoring the clinical relevance of antifungal‐biocide cross‐tolerance [46].

Virulence factors such as phospholipase, esterase, and haemolytic activities, along with aggregative phenotypes, may enhance *C. auris* persistence and biocide tolerance by strengthening biofilm integrity and limiting disinfectant penetration. Strong biofilm‐forming isolates often coexpress these traits, suggesting a synergistic protective effect mediated by the extracellular matrix, which can adsorb or neutralise biocides before exerting lethal activity. Aggregative phenotypes have also been linked to increased biofilm robustness and reduced susceptibility to agents like H_2_O_2_ and chlorhexidine [47].

In a study we conducted on this topic, 47 clinical Clade 1 isolates were tested against four disinfectants (triclosan, benzalkonium chloride, chlorhexidine and NaOCl), and most strains showed reduced susceptibility, particularly to triclosan and benzalkonium chloride. A key mechanism behind this tolerance is biofilm formation, which was observed in nearly all isolates [12]. The biofilm matrix can physically block biocides from reaching fungal cells and create a protective microenvironment, leading to reduced killing. Additionally, biofilm‐associated cells often upregulate efflux pumps, which actively remove toxic compounds and modify their cell membrane composition, decreasing biocide penetration. These adaptive responses are similar to mechanisms used for antifungal resistance and explain some cross‐resistance patterns observed between antifungal agents and biocides. For example, significant correlations were found between amphotericin B and triclosan MICs and between caspofungin and benzalkonium chloride MICs, suggesting shared resistance pathways. Furthermore, prolonged exposure to sub‐inhibitory concentrations of disinfectants may select for more tolerant strains. Overall, the study highlights that biocide tolerance in *C. auris* is not due to a single factor but results from combined mechanisms such as biofilm protection, efflux activity, and membrane adaptation, which together contribute to its persistence in hospital environments and make infection control more difficult [12].

## 5 Conclusions and Prospects

In summary, *C. auris* demonstrates a multifactorial tolerance to biocidal agents that parallels its antifungal resistance profile. Rather than relying on a single genetic determinant, its survival strategy combines biofilm‐mediated protection, efflux pump activation and membrane remodelling, allowing adaptation to a wide range of chemical stresses. The organism's ability to persist on medical surfaces, resist commonly used disinfectants and recover after sublethal exposure underscores the need for stringent infection control measures and optimised decontamination protocols. Future research should focus on identifying the specific regulatory networks and efflux transporters responsible for this tolerance, as well as evaluating combination strategies such as the concurrent use of chemical and physical disinfection methods to overcome environmental persistence. Understanding these mechanisms at the molecular level will be essential for developing evidence‐based guidelines to effectively control *C. auris* in healthcare environments.

## Author Contributions

Conceptualization, Ayse Kalkanci; methodology, Sidre Erganis, Ali Ozturk, and Elif Ayca Sahin; writing – original draft preparation, Sidre Erganis, Ali Ozturk, and Elif Ayca Sahin; writing – review and editing, Ayse Kalkanci. All authors have read and agreed to the published version of the manuscript.

## Funding

The authors have nothing to report.

## Consent

The authors have nothing to report.

## Conflicts of Interest

The authors declare no conflicts of interest.

## Data Availability

The data that support the findings of this study are available from the corresponding author upon reasonable request.
